# PlGF Reduction Compromises Angiogenesis in Diabetic Foot Disease Through Macrophages

**DOI:** 10.3389/fimmu.2021.736153

**Published:** 2021-09-29

**Authors:** Lingyan Zhu, Jieqi Qian, Yinan Jiang, Tianlun Yang, Qiong Duan, Xiangwei Xiao

**Affiliations:** ^1^ Department of Endocrinology, The First Affiliated Hospital of Nanchang University, Nanchang, China; ^2^ Department of Endocrinology, The Peoples Hospital of Yudu County, Ganzhou, China; ^3^ Department of Surgery, Children’s Hospital of Pittsburgh, School of Medicine, University of Pittsburgh, Pittsburgh, PA, United States; ^4^ Department of Cardiology, Xiangya Hospital, Central South University, Changsha, China; ^5^ Department of Cardiology, the First Affiliated Hospital of Nanchang University, Nanchang, China

**Keywords:** diabetic foot disease (DFD), placental growth factor (PlGF), vascular endothelial growth factor receptor 1 (VEGFR1), macrophages, angiogenesis

## Abstract

Diabetic foot disease (DFD) is a common and serious complication for diabetes and is characterized with impaired angiogenesis. In addition to the well-defined role of vascular endothelial growth factor (VEGF) -A and its defect in the pathogenesis of DFD, another VEGF family member, placental growth factor (PlGF), was also recently found to alter expression pattern in the DFD patients with undetermined mechanisms. This question was thus addressed in the current study. We detected attenuated PlGF upregulation in a mouse DFD model. In addition, the major cell types at the wound to express the unique PlGF receptor, VEGF receptor 1 (VEGFR1), were macrophages and endothelial cells. To assess how PlGF regulates DFD-associated angiogenesis, we injected recombinant PlGF and depleted VEGF1R specifically in macrophages by local injection of an adeno-associated virus (AAV) carrying siRNA for VEGFR1 under a macrophage-specific CD68 promoter. We found that the angiogenesis and recovery of the DFD were both improved by PlGF injection. The PlGF-induced improvement in angiogenesis and the recovery of skin injury were largely attenuated by macrophage-specific depletion of VEGF1R, likely resulting from reduced macrophage number and reduced M2 polarization. Together, our data suggest that reduced PlGF compromises angiogenesis in DFD at least partially through macrophages.

## Introduction

Vascular endothelial growth factor -A (VEGF-A), a potent endothelial cell mitogen, has been shown to play a substantial role in angiogenesis (1). VEGF is a heparin-bonded glycoprotein that has two specific receptors, VEGF receptor 1 (VEGFR1) and VEGF receptor 2 (VEGFR2) (2). Besides VEGF-A, VEGF family has 5 other members, among which placental growth factor (PlGF) is a critical one and has been shown to be associated with pathological angiogenesis (3).

Peripheral neuropathy and peripheral vascular disorders hallmark the pathology of DFD (4). The past studies have demonstrated defect in VEGF-A-mediated angiogenesis in DFD (5). Our lab has studied diabetes-associated angiogenesis for years (6–8). We have also shown that enhanced VEGF-A translation through genetic modulation of non-coding RNAs in human mesenchymal stem cells (MSCs) promotes the secretion of VEGF-A by MSCs to improve the angiogenesis and recovery of the injury at the wound site in DFD (9, 10). Recently, we have shown that PlGF plays a critical role in gestational beta-cell growth (11), likely as a potent growth factor that regulates the crosstalk between trophic macrophages and pancreatic beta-cells (11). Interestingly, upregulation of PlGF has recently been shown during wound healing, and the levels of PlGF were reduced in DFD patients (12), and in mouse models for DFD (13). In another study that investigated the role of PlGF in diabetic wound healing, the effects of PlGF on fibroblasts but not on macrophages were investigated (14). Since the major cell types that harbor the unique PlGF receptor VEGFR1 are macrophages, endothelial cells and fibroblasts (2), the exact molecular mechanisms underlying PlGF-regulated angiogenesis in DFD seems warrant further investigation, which was thus addressed in the current study.

We detected attenuated PlGF upregulation in a mouse DFD model. In addition, the major cell types at the wound to express the unique PlGF receptor, VEGFR1, were macrophages and endothelial cells. To assess how PlGF regulates DFD-associated angiogenesis, we injected recombinant PlGF and depleted VEGF1R specifically in macrophages by local injection of an adeno-associated virus (AAV) carrying siRNA for VEGFR1 under a macrophage-specific CD68 promoter. We found that the angiogenesis and the recovery of the DFD were both improved by PlGF injection but were largely attenuated by macrophage-specific depletion of VEGF1R, likely resulting from reduced macrophage recruitment and reduced M2 polarization.

## Materials and Methods

### Protocol Approval

All the experimental protocols, animal surgery and treatments applied in the current study have been approved by the research committee and Institutional Animal Care and Use Committee at the First Affiliated Hospital of Nanchang University, respectively. The experiments were carried out in accordance with the approved guidelines. For each animal experiment, a power calculation (p<0.05) was performed to determine exactly sufficient number of mice to allow the observed effects to be legitimate. An allocation concealment method was used to provide randomization in allocating experimental units to control and treatment groups. The use of the inbred littermate mice in a specified experiment ensured that the potential confounders were minimized. No criteria were used for excluding animals (or experimental units) during the experiment, and no data were excluded during the analysis.

### Induction of Diabetes, DFD and AAV Transplantation

Randomization and blind assessment were used in all animal studies. Diabetes was induced in 12-week-old C57/BL6 mice (male and female are evenly distributed in each group, SLAC Laboratory Animal, Shanghai, China) by single intraperitoneal injection of 160 mg/kg streptozotocin (STZ) in 100µl normal saline after a 12-hour fasting, as described before (15). This dose used in fasting wildtype mice results in 100% development of diabetes, since STZ specially goes into beta-cells through glucose transporter 2 (Glut2) on beta-cells. Fasting is critical since circulating glucose can compete with STZ for Glut2 to compromise the effects of STZ. The control mice received equal volume of the normal saline, the solvent to dissolve STZ. One week later, all mice that had received STZ became hyperglycemic (fasting blood glucose >=350mg/dl). If any mice did not develop hyperglycemia (fasting blood glucose >=350mg/dl) one week after STZ, they were supposed to be excluded from this study. The STZ-treated mice then underwent a surgery to create a 6 mm-diameter full-thickness wound on the dorsal midline with a biopsy punch, with/without orthotopic injection with 100µl AAV viruses [10^11^ genome copy particle (GCP)/ml], as described (16). Meanwhile, the wounds of the experimental mice were treated with 50 µl recombinant PlGF (10 µg, Ab207150, Abcam, Cambridge, MA, USA) or an equivalent volume of saline solution a Hamilton’s syringe under the clot covering the wound at a frequency of twice per week. Fasting blood glucose were performed at 10am after a three-hour fasting period and assessment of beta-cell mass was done as described (17, 18). For vessel density measurement, tomato-lectin (50 µl, Vectorlabs, Burlingame, CA USA) was injected to the mice through the tail vein 10 minutes before sacrifice. The vessel density was determined bases on the percentage of lection-positive area in total measured tissue area, as described before (10).

### Production of AAVs

Transfection of human embryonic kidney 293 cells by AAV serotype 6 vectors were applied by as described before (19, 20). The backbone plasmid and the plasmid containing human CD68 promoter were both obtained from an Addgene plasmid (#32395 (21) and #34837 (22), Watertown, MA, USA). The sequence for siRNA for VEGFR1 was 5’-CCAGACACTGCATCCAA-3’, and the control scramble sequence was 5’-GCATTAACTAAAGGCUGCC-3’. The Transfection was performed with Lipofectamine 3000 reagent (Invitrogen, CA, Carlsbad, USA). Purification and titration of AAV vectors were performed with standard procedure and a dot-blot assay, respectively, as described before (19).

### Flow Cytometry

The injured skin tissue was dissected out and digested with 0.25% trypsin for 35 minutes to obtain a single cell fraction for flow cytometry. The flow cytometry-based cell purification was based on direct fluorescence from GFP (AAVs-originated) and Tomato (Lectin-originated) as well as immunofluorescence from VEGFR1 (by a PE-cy7-conjugated anti-VEGFR1 antibody, Becton-Dickinson Biosciences, San Jose, CA, USA) or CD68 (by a BV421-conjugated anti-CD68 antibody) or CD31 (by an APC-conjugated anti-CD68 antibody), as described before (15). The flow cytometry data were analyzed by Flowjo (version 11.0, Flowjo LLC, Ashland, OR, USA).

### Quantitative Real-Time PCR (RT-qPCR)

A RNeasy mini kit (Qiagen, Germantown, MD, USA) was used to extract RNA. Quantitative real-time PCR (RT-qPCR) were performed using commercial primers ordered from Qiagen. Data were collected and analyzed using 2-△△Ct method. Values of genes were obtained by sequential normalization against the internal control GAPDH and the corresponding experimental controls.

### ELISA

The extracted protein by RIPA buffer was subjected to mouse PlGF-2 (MP200) and VEGFR1 (MVR100) enzyme-linked immunosorbent assay (ELISA; R&D System, Los Angeles, CA, USA), as described before (10). The microplate was read at 450 nm within 30 minutes. The wavelength correction was set to 540 nm or 570 nm.

### Histology and Immunostaining

Ten minutes after tomato-lectin infusion through the tail vein, the mice were sacrificed. The pancreas and the injured skin were dissected out and fixed in 4% paraformaldehyde (PFA, Sigma-Aldrich, St. Louis, MO, USA) for 6 hours. After incubation in 30% sucrose for 24 hours, the samples were frozen and embedded. Fluorescent immunostaining was done as described before (10). Tomato-lectin was detected by direct red fluorescence and virus-transduced macrophages were detected by GFP. Immunofluorescent staining for glucagon and insulin was performed with a monoclonal antibody against glucagon (Ab10988, Abcam) and a guinea pig polyclonal antibody against insulin (Ab7842, Abcam), respectively. Hoechst 33342 (HO, Sigma-Aldrich) was applied to stain the nucleus.

### Bioinformatics

Gene Expression Omnibus (GEO, http://www.ncbi.nlm.nih.gov/geo/) was used to obtain data for bioinformatic analysis (23). The data surf applied “diabetic foot” OR “diabetic foot disease “ AND “Homo sapiens”. After a careful assessment for relatedness, two gene expression profiles (GSE80178 and GSE134431) were selected (24). The GEO2R online analysis tool and “DESeq2” R package were applied for determining the differentially expressed genes (DEGs) and calculation for P-value, adjusted P-value and logFC. PlGF levels in both series were obtained and analyzed the difference among groups. Pathway enrichment analyses of DEGs were performed at Metascape (http://metascape.org).

### Statistical Analysis

Individual values were represented in the figures. Statistical analysis was carried out using a one-way analysis of variance (ANOVA) test followed by the Fisher’s Exact Test (GraphPad Software, version 9, Inc. La Jolla, CA, USA). A value of p<0.05 was considered statistically significant.

## Results

### PlGF Upregulates During Foot Wound Healing and This Upregulation Is Compromised in Diabetes

**Figure 1:**
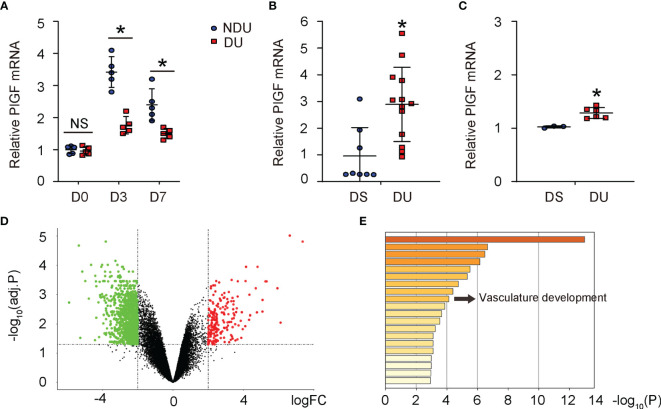
PlGF upregulates during foot wound healing and this upregulation is compromised in diabetes. **(A)** ELISA for PlGF in diseased foot tissue from a mouse model for DFD before induction of ulcer (day 0) and day 3 and day 7 after induction of DFD in mice. DU: diabetic ulcer. NDU: non-diabetic ulcer. **(B, C)** PlGF mRNA levels in diabetic foot skin (DS, equal to DU day 0) *versus* diabetic foot ulcer (NDU, equal to DU day 3) from human DFD dataset GSE134431 **(B)** and GSE80178 **(C)**. **(D)** A hot map to show difference in gene expression from DS *versus* DU in GSE80178. **(E)** A pathway analysis to show difference in enriched pathway between DS and DU in GSE80178. *p < 0.05. NS, non-significant.

Since recent studies revealed a possible role of PlGF in DFD, we analyzed tissue PlGF levels at day 3 and day 7 after induction of DFD in mice (9, 10). PlGF levels in diabetic foot skin (or diabetic ulcer (DU) at day 0), did not differ from non-diabetic controls (or non-diabetic ulcer (NDU) at day 0) (**Figure 1A**). PlGF levels increased at both day 3 and day 7 after ulcer induction, regardless of the diabetic status (**Figure 1A**), which was consistent with the analysis on 2 human DFD datasets (GSE134431, **Figure 1B**; GSE80178, **Figure 1C**). However, the overall increases in PlGF levels were significantly attenuated in diabetes (**Figure 1A**). The analysis on human DFD dataset GSE80178 also showed significant difference in gene expression (**Figure 1D**) and vasculature development pathway (**Figure 1E**). Together, these data highlight the importance of angiogenesis and suggest a possible role of PlGF in the pathological process of DFD.

### VEGFR1 Is Expressed in Macrophages, Endothelial Cells and Fibroblasts in DFD Tissue

**Figure 2:**
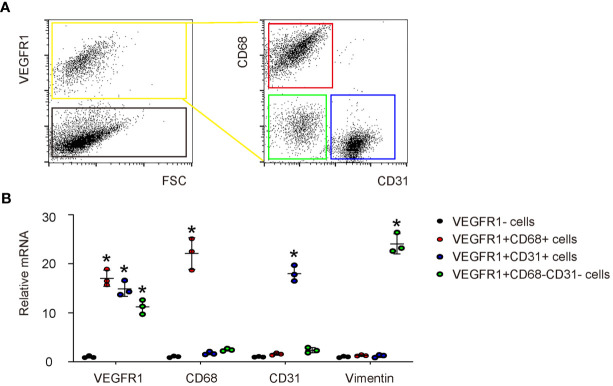
VEGFR1 is expressed in macrophages, endothelial cells and fibroblasts in DFD tissue. **(A)** A representative flow chart to show digestion of day 3 DFD tissue into single cell population and a subsequent sorting of VEGFR1+ cells *versus* VEGFR1- cells by flow cytometry. Afterwards, the VEGFR1+ cells were sorted for CD68 and CD31 to further separate VEGFR1+ cells into VEGFR1+CD68+ cells (macrophages), VEGFR1+CD31+ cells (endothelial cells), and VEGFR1+CD68-CD31- cells (VEGFR1+ cells that are not macrophages or endothelial cells). **(B)** RT-qPCR for VEGFR1, CD68, CD31 and vimentin on the sorted 4 cell fractions. *p < 0.05.

Next, we investigated the role of PlGF in angiogenesis and the wound recovery in DFD. Since PlGF has a unique receptor, VEGFR1, we analyzed the cell types that harbor VEGFR1 to respond to PlGF at the wound. In a previous publication, macrophages, endothelial cells and fibroblasts have been shown to express VEGFR1 (2). To confirm it, day 3 injured skin tissue was digested into single cell population and then sorted for VEGFR1+ cells *versus* VEGFR1- cells by flow cytometry (**Figure 2A**). Afterwards, the VEGFR1+ cells were further sorted for CD68 (a marker for macrophages) and CD31 (a marker for CD31). Therefore, the VEGFR1+ cells were in addition separated as VEGFR1+CD68+ cells (macrophages), VEGFR1+CD31+ cells (endothelial cells), and VEGFR1+CD68-CD31- cells (VEGFR1+ cells that are not macrophages or endothelial cells) (**Figure 2A**). We found that among all VEGFR1+ cells, either CD68+ cells or CD31+ cells are much more than CD68-CD31- cells, suggesting that most VEGFR1+ cells are either macrophages or endothelial cells (**Figure 2A**). RT-qPCR was then performed on the sorted cells, showing significantly higher VEGFR1 in all 3 VEGFR1+ populations than VEGFR1- population, significantly higher levels of CD68 in VEGFR1+CD68+ cell population than the other 3 cell populations, significantly higher levels of CD31 in VEGFR1+CD31+ cell population than the other 3 cell populations, and significantly higher levels of Vimentin (a marker for fibroblasts) in VEGFR1+CD68-CD31- cell population than the other 3 cell populations (**Figure 2B**), with the latter suggests that most fibroblasts are in the VEGFR1+CD68-CD31- cell population. Compared with results from **Figure 2A**, fibroblasts may represent a relatively smaller cell population that expresses VEGFR1.

### Generation of AAVs That Deplete VEGFR1 Specifically in Macrophages

**Figure 3:**
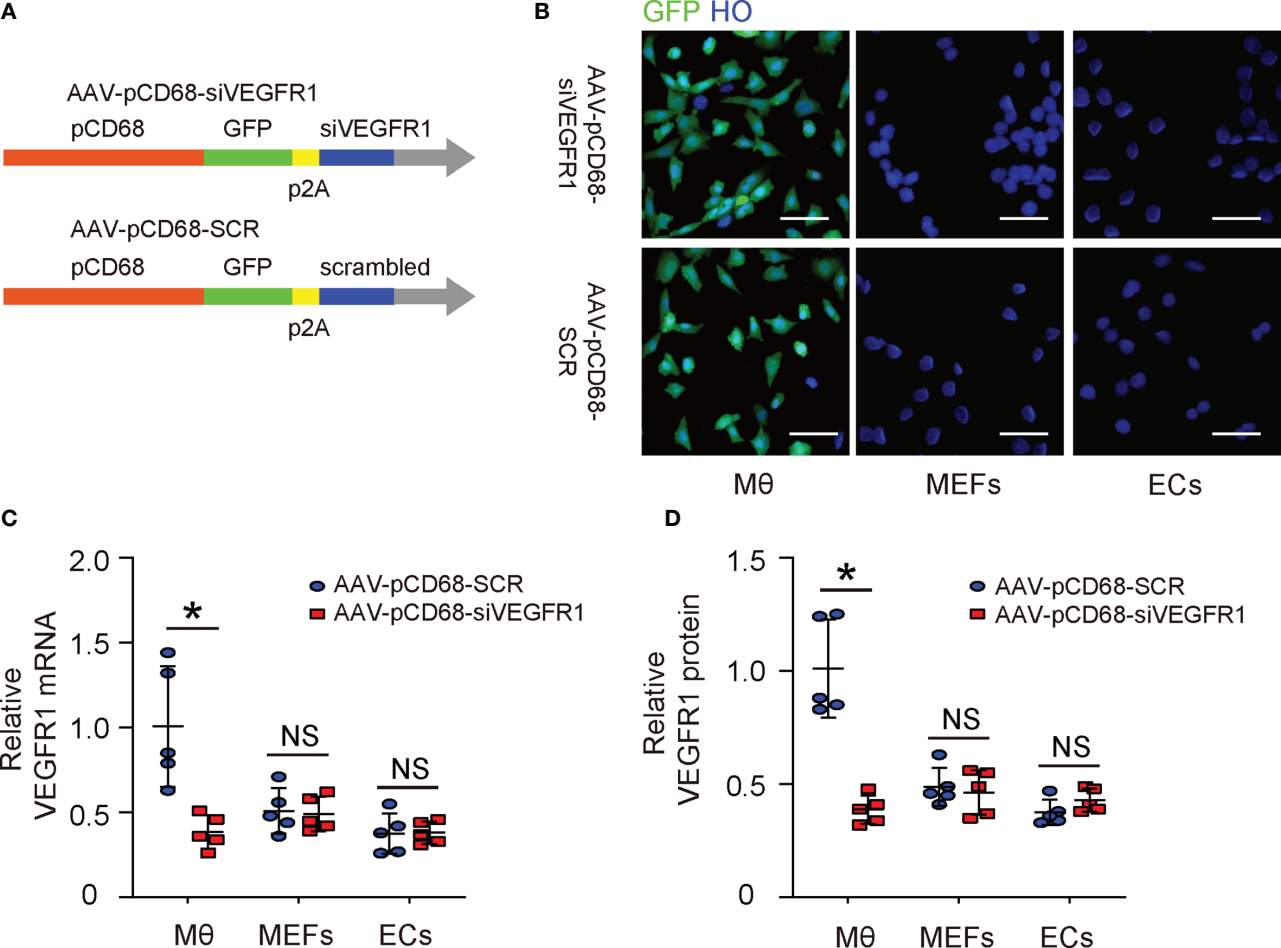
Generation of AAVs that deplete VEGFR1 specifically in macrophages. **(A)** Schematic of an AAV carrying siRNA for VEGFR1 under a macrophage-specific CD68 promoter (AAV-pCD68-siVEGFR1) and control AAV-pCD68-SCR. **(B)** Isolated macrophages (Mθ), endothelial cells (ECs) and mouse embryonic fibroblasts (MEFs) were infected with AAV-pCD68-siVEGFR1 or control AAV-pCD68-SCR, shown by representative immunofluorescent images. **(C, D)** RT-qPCR **(C)** and ELISA **(D)** for VEGFR1 in infected Mθ, ECs and MEFs. *p < 0.05. NS, non-significant. Scale bars are 20 µm.

The effects of PlGF on fibroblasts but not on macrophages during tissue repair have been investigated (14). Since macrophages are known to play a critical role in angiogenesis, and since we detected a great number of macrophages among all VEGFR1+ cells in the mouse DFD model (**Figure 2A**), here we focused on macrophages. We generated an AAV carrying siRNA for VEGFR1 under a macrophage-specific CD68 promoter (AAV-pCD68-siVEGFR1), which allows specific depletion of VEGFR1 in macrophages and allows us to assess the effects of PlGF on angiogenesis and recovery of the DFD injury through macrophages (**Figure 3A**). To ensure the specificity of this construct, isolated macrophages, endothelial cells and mouse embryonic fibroblasts (MEFs), 3 cell types that express VEGFR1, were infected with either AAV-pCD68-siVEGFR1 or control AAV-pCD68-SCR. We found that CD68 promoter allowed effective transduction of macrophages, but not endothelial cells or MEFs, based on the expression of the GFP reporter (**Figure 3B**). The levels of VEGFR1 in the infected cells were analyzed by RT-qPCR, showing significant reduction in VEGFR1 mRNA in AAV-pCD68-siVEGFR1-transduced macrophages, while the VEGFR1 mRNA in either endothelial cells or MEFs remained unchanged (**Figure 3C**). Similar results were obtained when VEGFR1 protein was analyzed by ELISA (**Figure 3D**). Thus, AAV-pCD68-siVEGFR1 specifically depletes VEGFR1 in macrophages.

### PlGF With/Without Macrophage-Depletion of VEGFR1 Does Not Affect Diabetes

**Figure 4:**
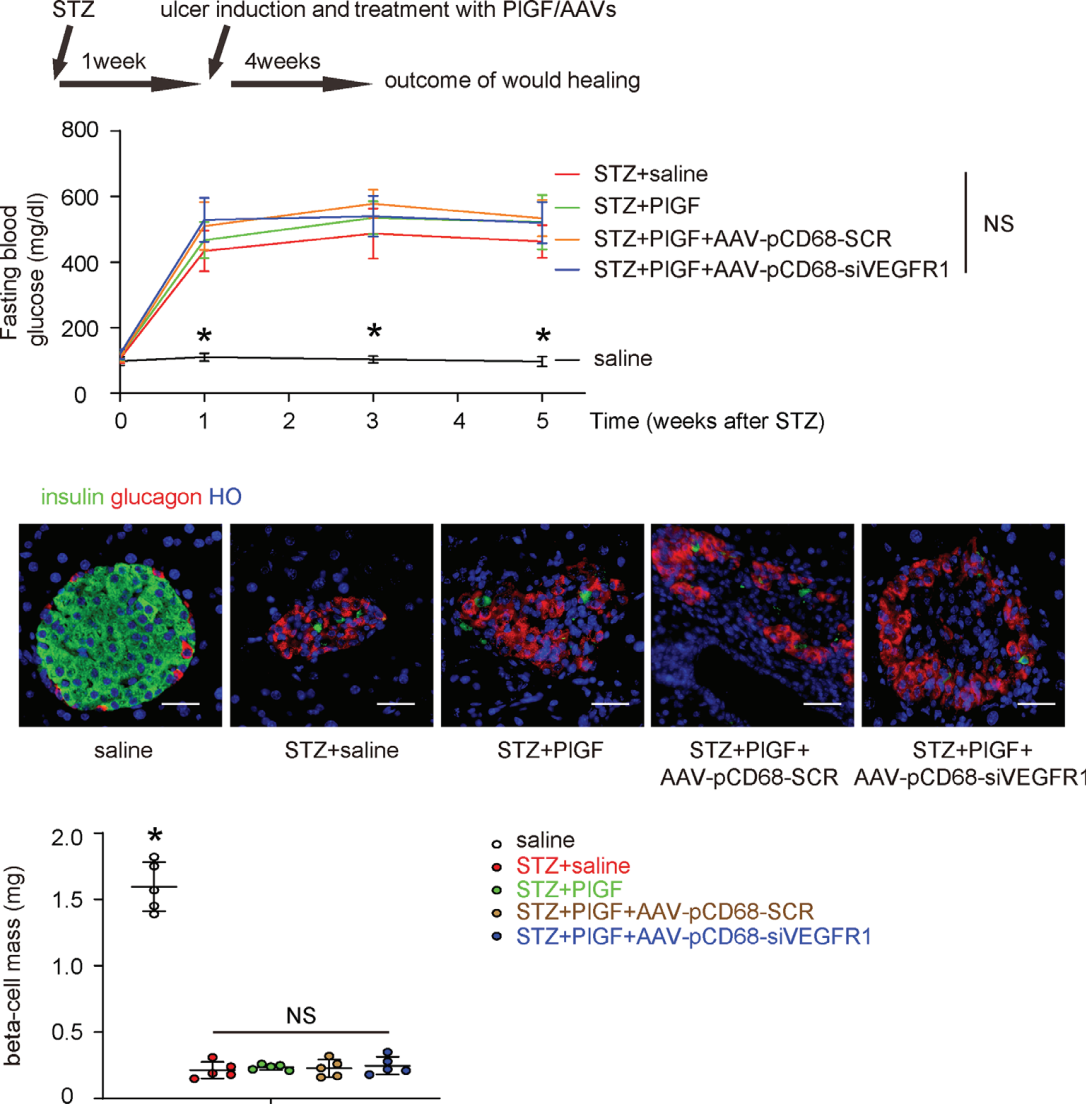
PlGF with/without macrophage-depletion of VEGFR1 does not affect diabetes. **(A)** Schematic of a mouse model for DFD, in which C56/BL6 mice received STZ to induce diabetes and then a surgical ulcer at dorsal midline after one week. At the time of surgical ulcer induction, PlGF or saline was orthotopically injected biweekly while AAVs were given locally only once, immediately after ulcer induction. In another 4 weeks, the mice were sacrificed and analyzed. **(B)** Fasting blood glucose. **(C)** Representative immunofluorescent images for glucagon and insulin in mouse pancreas. **(D)** Beta-cell mass at sacrifice. *p < 0.05. NS, non-significant. Scale bars are 20 µm.

The effects of PlGF on DFD and the role of macrophages were then assessed in a mouse model for DFD, in which C56/BL6 mice received STZ to induce diabetes and then received a surgical ulcer at dorsal midline after one week. At the time of surgical ulcer induction, PlGF or saline was orthotopically injected biweekly while AAVs were given locally to the wound only once, immediately after ulcer induction. We have previously shown that the AAV-mediated transduction *in vivo* can sustain much longer than the one month’s experimental time window (25–27). After another 4 weeks, the mice were sacrificed and analyzed (**Figure 4A**). We found that STZ treatment induced irreversible hyperglycemia in mice, while any treatments or treatment combinations neither correct hyperglycemia (**Figure 4B**), nor alter beta-cell mass (**Figures 4C, D**). These data suggest that the sustained hyperglycemia induced by STZ resulted from loss of beta-cells rather than from other reasons, i.e. insulin resistant, in which hyperglycemia is developed while the beta cell number can remain normal or even increase at the early stage (28). Moreover, PlGF treatment with/without macrophage-depletion of VEGFR1 does not affect diabetes.

### PlGF Promotes Wound Healing and Angiogenesis, Which Are Attenuated by Macrophage-Depletion of VEGFR1

**Figure 5:**
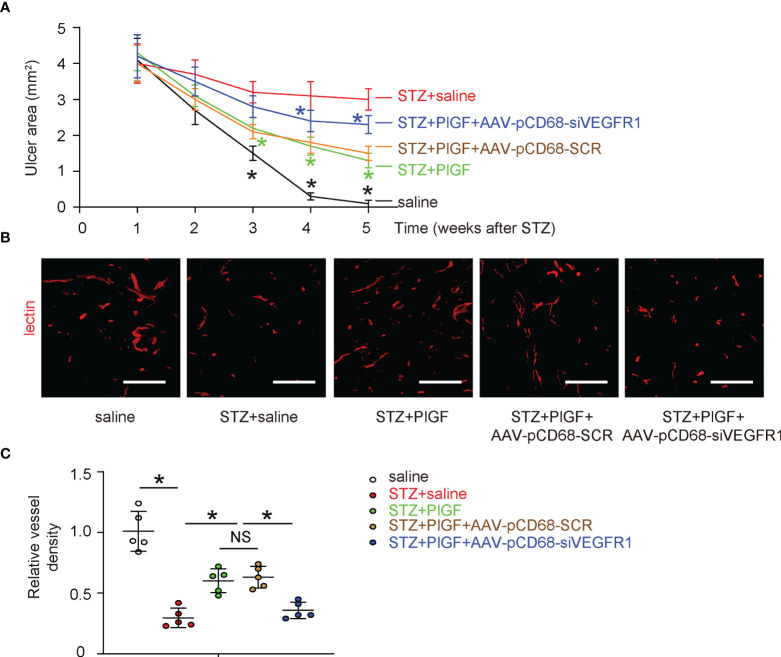
PlGF promotes wound healing and angiogenesis, which are attenuated by macrophage-depletion of VEGFR1. **(A)** The effects of PlGF through macrophages on wound healing of DFD were examined. **(B, C)** Angiogenesis was analyzed at the end of the experiment by vessel density (5 weeks after STZ or 4 weeks after PlGF/AAVs), shown by representative fluorescent images **(B)** and by quantification **(C)**. *p < 0.05. In panel **(A)**, black star: STZ *vs* saline, green star: STZ+PlGF *vs* STZ, blue star: STZ+PlGF+AAV-pCD68-siVEGFR1 *vs* STZ+PlGF+AAV-pCD68-SCR. NS, non-significant. Scale bars are 100 µm.

Next, we examined the effects of PlGF through macrophages on the wound healing in DFD. We found that the wound was completely cured at 4 weeks after ulcer induction in saline-treated normoglycemic mice (saline) (**Figure 5A**). The ulcers did not cure in all STZ-treated mice. However, better recovery was detected in diabetic mice treated with PlGF (**Figure 5A**). This improvement of wound recovery by PlGF, was however, abolished by macrophage-depletion of VEGFR1 (**Figure 5A**). Angiogenesis was analyzed at the end of the experiment by vessel density (5 weeks after STZ or 4 weeks after PlGF/AAVs). We found that the vessel density was significantly reduced by STZ treatment in the injured tissue (**Figures 5B, C**). However, improved angiogenesis was detected in diabetic STZ-mice treated with PlGF (**Figures 5B, C**). This improved angiogenesis by PlGF was also abolished by macrophage-depletion of VEGFR1 (**Figures 5B, C**). Thus, PlGF promotes wound healing and angiogenesis, which are likely through VEGFR1 on macrophages.

### Macrophage-Depletion of VEGF1R Reduces the Number of Macrophages and Reduces M2 Polarization

**Figure 6:**
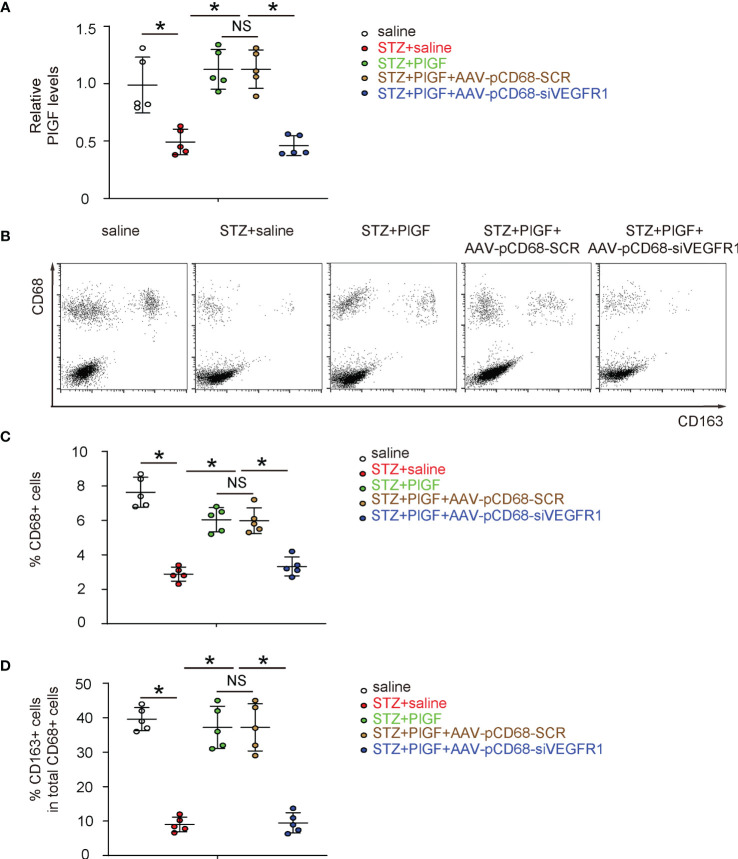
Macrophage-depletion of VEGF1R reduces macrophage number and reduces M2 polarization. **(A)** ELISA for PlGF levels in tissue from all experimental groups. **(B)** Representative flow charts for analysis and sorting for CD68 and CD163. **(C, D)** Quantification of CD68+ macrophage percentage **(C)** and CD163+ cells in total CD68+ cells **(D)**. *p < 0.05. NS, non-significant.

Since we found that PlGF-induced improvement in angiogenesis and recovery of DFD was largely attenuated by macrophage-specific depletion of VEGF1R, we examined the changes in macrophages in response to PlGF and VEGFR1 depletion. First, PlGF in tissue was assessed in all experimental groups, showing significant reduction by STZ and significant increase by PlGF (**Figure 6A**). Surprisingly, PlGF levels were reduced by macrophage-depletion of VEGFR1 (**Figure 6A**). Next, macrophages (CD68+) and their M2 (CD68+CD163+) *versus* M1 (CD68+CD163-) polarization were analyzed by flow cytometry (**Figures 6B–D**). Interestingly, we found that STZ not only reduced the number of macrophages, but also reduced their M2 polarization (**Figures 6B–D**). Moreover, PlGF not only increased the number of macrophages, but also enhanced their M2 polarization (**Figures 6B–D**). Finally, the effects of PlGF on both macrophage number and their M2 polarization were both attenuated by macrophage-depletion of VEGFR1 (**Figures 6B–D**).

## Discussion

Wound healing is orchestrated by a number of biological events that include tissue hemostasis, local inflammation, cell proliferation, and structure remodeling, during which both macrophages and vascular endothelial cells play a pivotal role (29). Endothelial cells are activated by hypoxia and increase vascular permeability to recruit inflammatory cells including macrophages to the wound site (29). The interaction between endothelial cells and macrophages alters the phenotypes of each other to allow not only the oxygen and nutrient transit from circulation to promote cell proliferation and differentiation, but also the production and release of growth factors like proangiogenic factors from macrophages to affect the formation of new capillaries through angiogenesis (30). Among these factors, the most potent one is VEGF-A, which has been known as the most important angiogenic factor in wounds (31). However, recent studies have demonstrated a previously neglected role of PlGF in angiogenesis, especially in diabetes (14), as it is likely more important in pathological angiogenesis (32).

Here, we showed reduced PlGF in DFD, and correction of PlGF could improve the impaired angiogenesis and wound recovery of DFD, suggesting that PlGF, in addition to VEGF-A, may play a substantial role in angiogenesis under a diabetic status. Unlikely VEGF-A, which has two receptors VEGFR1 and VEGFR2, PlGF functions exclusively through VEGFR1 (33). Interestingly, both VEGFR1 and VEGFR2 are expressed by endothelial cells, while previous studies have shown that VEGFR1 on endothelial cells appears to have a higher affinity but a lower potency with VEGF-A, compared to VEGFR2 (34). Thus, the PlGF/VEGFR1 signaling may play a negative role in endothelial cells during angiogenesis. That is the reason that we did not investigate the effect of PlGF on endothelial cells in the current study. On the other hand, PlGF/VEGFR1 signaling is critical for macrophages that harbor VEGFR1 but not VEGFR2 (35). Specially, PlGF has been shown to induce polarization of macrophage to a M2-like subtype, which is important for cell proliferation, regeneration and tissue remodeling, as we showed before (27). This concept has been well confirmed in the current study, as we detected potential increases in the number of macrophages and their M2 polarization induced by PlGF. Macrophages that have undergone a M2 polarization may release PlGF to bind VEGFR1 on their own to create an autocrine positive regulatory loop to amplify the effect, as reported before (36), which could explain why CD68+ cell number and CD163+ M2 macrophage percentage in total macrophages were both reduced by macrophage-depletion of VEGFR1 (**Figures 6B–D**). Indeed, since VEGFR1 is the only receptor for PlGF, PlGF secreted by injured tissue or by the experimental administration (recombinant PlGF injeciton) can recruit macrophages *via* their VEGFR1, which is a typical chemokine effect. On the other hand, siRNA for VEGFR1 under the control of a macrophage-specific CD68 promoter allows exclusive transduction of macrophages by siVEGFR1, resulting in the loss of the response of macrophages to PlGF. Hence, the PlGF/VEGFR1 regulatory axis is abolished to prevent a positive effect on the macrophage polarization and migration, during which macrophages are induced to produce and secrete PlGF to create an autocrine effect to amplify this effect (36). Given the relative lower effect of fibrosis on angiogenesis and the unique negative effect of PlGF on angiogenesis through VEGFR1 on endothelial cells, the importance of PlGF to the diabetic angiogenesis may largely result from its effect on macrophages, as shown in this study.

The finding in this study is well in line with some clinical trials of autologous cell therapy based on peripheral blood mononuclear cells implant (PBMNC) in critical limb ischemia patients. First, autologous PBMNC implant is shown to increase angiogenesis and wound healing in critical limb-ischemic diabetic patients and to reduce amputations (37). Second, PBMNC, but not bone marrow mononuclear cells or bone marrow mesenchymal cells, has been found to reduce limb amputation in critical limb-ischemic diabetic patients (38). Third, the autologous PBMNC implants in unhealing wound increase both the macrophage number, VEGF paracrine release and M2 polarization of macrophages (39). Moreover, a recent paper showed that some biomaterials increase and accelerate healing in diabetic foot inducing M2 polarization (40).

The trophic factors produced and released by M2-polarized macrophages, were however, not determined in the current study. In future, RNA-seq analysis could be performed to figure out the growth factors important to macrophage-enhanced angiogenesis. Another limitation of the current study is the use of the rodent model, which only partially mimics the DFD in humans. However, it provides important information regarding the underlying molecular mechanisms that can be translatable to human research. Future approaches may assess the role of PlGF and macrophages in non-human primates, which could help to extrapolate the findings in the current study to the therapy in human patients.

To summarize, here we used specific gain-of-function and loss-of-function experiments to demonstrate a role of PlGF in DFD, which is largely mediated by macrophages and their polarization. Our study should provide strong evidence for investigating DFD through novel therapeutic targets.

## Data Availability Statement

The original contributions presented in the study are included in the article/supplementary material. Further inquiries can be directed to the corresponding authors.

## Ethics Statement

The animal study was reviewed and approved by the First Affiliated Hospital of Nanchang University.

## Author Contributions 

LZ, JQ, YJ, TY, DD and XX are responsible for data acquisition and analysis. JQ and XX performed bioinformatics analysis. LZ and XX are responsible for study conception and design, data acquisition and analysis. XX wrote the manuscript and all authors have read the manuscript and agreed with the publication. LZ and XX are responsible for funding and are the guarantee of the study. All authors contributed to the article and approved the submitted version.

## Funding

This work was supported by grants from an Assistant Professor Startup from University of Pittsburgh (To XX), National Natural Science Foundation of China (NO 82160155 and 81860153 to LZ, NO 81770358 to TY, China Traditional Chinese and Western Medicine Committee Hehuang Scientific Research Fund (NO 2019005 to LZ), Key Project of Natural Science Foundation of Jiangxi Province (NO 20202ACBL216007 to LZ), Education and Teaching Program of Nanchang University (NO NCUYJSJG-2021-068 and 20190054 to LZ) and Science and Technology Plan of Health Commission of Jiangxi Province (No 20203106 to LZ).

## Conflict of Interest

The authors declare that the research was conducted in the absence of any commercial or financial relationships that could be construed as a potential conflict of interest.

## Publisher’s Note

All claims expressed in this article are solely those of the authors and do not necessarily represent those of their affiliated organizations, or those of the publisher, the editors and the reviewers. Any product that may be evaluated in this article, or claim that may be made by its manufacturer, is not guaranteed or endorsed by the publisher.
